# Synapse loss and progress of Alzheimer’s disease -A network model

**DOI:** 10.1038/s41598-019-43076-y

**Published:** 2019-04-25

**Authors:** G. Kashyap, D. Bapat, D. Das, R. Gowaikar, R. E. Amritkar, G. Rangarajan, V. Ravindranath, G. Ambika

**Affiliations:** 1 Indian Institute of Science Education and Research(IISER) Pune, Pune, 411008 India; 2 Indian Institute of Science, Center for Neuroscience, Bangalore, 560012 India; 3 Physical Research Laboratory, Ahmedabad, 380009 India; 4 Indian Institute of Science, Department of Mathematics, Bangalore, 560012 India; 5 Indian Institute of Science Education and Research(IISER) Tirupati, Tirupati, 517507 India

**Keywords:** Network models, Complex networks

## Abstract

We present observational evidence from studies on primary cortical cultures from AD transgenic mice, APPSwe/PS1ΔE9 (APP/PS1) mice, for significant decrease in total spine density at DIV-15 and onward. This indicates reduction in potential healthy synapses and strength of connections among neurons. Based on this, a network model of neurons is developed, that explains the consequent loss of coordinated activity and transmission efficiency among neurons that manifests over time. The critical time when structural connectivity in the brain undergoes a phase-transition, from initial robustness to irreparable breakdown, is estimated from this model. We also show how the global efficiency of signal transmission in the network decreases over time. Moreover, the number of multiple paths of high efficiency decreases rapidly as the disease progresses, indicating loss of structural plasticity and inefficiency in choosing alternate paths or desired paths for any pattern of activity. Thus loss of spines caused by *β*-Amyloid (A*β*) peptide results in disintegration of the neuronal network over time with consequent cognitive dysfunctions in Alzheimer’s Disease (AD).

## Introduction

Alzheimer’s disease (AD) is one of the most devastating disorders afflicting the older population^1,2^, and aging is one of the major risk factors for dementia, including AD. Recent advancements in medical health-care have increased the longevity of human life, consequently increasing the incidence of AD^3,4^. As of now, neither cure nor therapeutic approaches are available. Several studies have shown that accumulation of *β*-Amyloid-42 (A*β*) and reduction in the volume of the hippocampus occurs in human subjects years before the appearance of clinical symptoms^5–9^. Recent studies conducted using [^18^F]FDG PET (^18^F - fluorodeoxyglucose Positron Emission Tomography) have indicated that loss of neuronal function can be detected in human subjects decades before the onset of AD^10^. Thus decreased utilization of 2-deoxy-2-[fluorine-18]fluoro-D-glucose (FDG), seen by PET imaging in human subjects, occurs 2–3 decades before the frank appearance of AD symptoms^11,12^ and is indicative of synaptic dysfunction. Therefore, the study of structural and biochemical changes that precede cognitive impairment is of paramount importance as it may enable us to intervene before the brain undergoes significant irreversible damage. In this context, we emphasise the need to study the pre-symptomatic or prodromal phases of AD that can help to identify probable cases earlier. So also, studies exploring the mechanisms underlying disease pathogenesis are important. Such studies can lead to development of therapeutic approaches that could potentially delay the onset of AD or alter the clinical course.

It is reported that synaptic dysfunction is one of the major contributors to AD pathogenesis^13–22^. Studies using transgenic mice, harbouring mutations known to cause familial AD in humans, have provided evidence to support the theory that cognitive dysfunction observed in these mice correlates to loss of spines^5,6,13,17,23^. It is reported that F-actin (fibrillar actin) is the major cytoskeletal protein that determines the shape of spines and exposure to A*β* leads to loss of F-actin by depolymerization to G-actin (globular actin), thus contributing to the collapse of spines^23^. The trigger and mechanisms underlying these structural changes, however, remain poorly understood. Further, it is not clear whether these changes occur in the prodromal phase, and if so, whether they are predictive of the impending impairment of synaptic activity and ensuing cognitive deficits. Also how changes at the synapse level manifest as dysfunctions at the neuronal network level is not so far properly modelled or studied.

We hypothesize that changes in synapses induced by A*β* can reduce the innate ability of neurons and alter the efficacy of transmission in response to specific activity patterns, resulting in failure to respond with the normally required pattern for any specific activity. We try to understand the structural and functional changes in neuronal connections that can help in an early diagnosis and therapy of AD.

In this context we note the transmission of electrical signals from neuron to neuron, connected in complex networks and circuits, is central to brain function^24–27^. The network connectivity determines the coordinated generation of an output from ensembles of neurons to execute complex cognitive functions including learning and memory. The synaptic connectivity between neurons is dynamic and exhibits plasticity, which is the fundamental process observed in learning and memory^28,29^. The cellular and molecular basis of synaptic transmission, at the level of neurons is reasonably well understood^30–32^. However, the role of connectivity in the transmission of signals at the neuronal network level is not fully explored.

For the study of neurons at the network level, we consider the fact that the output of the neuronal network is determined by the integrated activity of all individual neurons. Also the input signals to each neuron is the integrated one from all of its connections and this will be subsequently transmitted to many neurons. So changes in one synapse affecting its efficiency and local synaptic plasticity can affect the performance of several neurons linked to it. Hence, the efficiency of transmission at the network level and the network connectedness are important measures that will indicate the extent to which A*β*-induced neurodegeneration at individual synapses affects the coordination of the global activity of neuronal networks. In the model presented here, using the framework of complex networks, we establish how spine loss effectively reduces the connectivity and hence deprives the neuronal networks of their normal functions over time.

## Results

### Spine density is reduced in primary neurons derived from APP/PS1 mice

**Figure 1:**
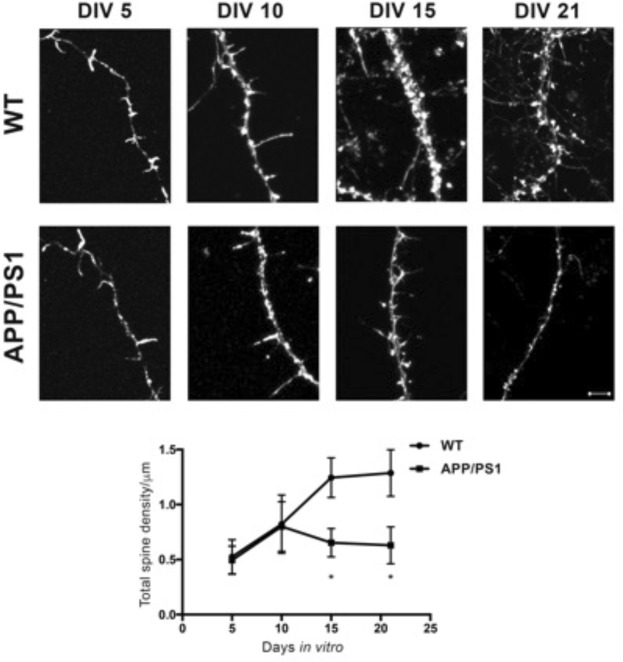
Representative confocal images of dendritic spines of neurons from WT and APP/PS1 mice at DIV (days *in-vitro*)-5, DIV-10, DIV-15 and DIV-21. Scale bar is 5 *μ*m. Top - Quantification for total spine density at DIV-5, 10, 15 and 21. Bottom - Total spine density of wild-type (WT) and APP/PS1 groups is similar at DIV-5 and 10. Significant spine loss is observed at DIV-15 and is sustained till DIV-21. Data is represented as Mean + SEM (n = 30 neurites per group) and statistical significance: *p < 0.0001 for WT versus APP/PS1 (Two-way RM ANOVA followed by Bonferroni post-hoc test).

In order to study changes in spine density, we used primary cortical cultures from APPSwe/PS1ΔE9 (APP/PS1) and wild type (WT) mice. Spine density was measured in cultured primary neurons derived from the cortex of WT and APP/PS1 mice on post-natal day 1. The cells were maintained in culture for varying time periods (5, 10, 15, and 21 days *in-vitro*; DIV), the cells were stained with phalloidin (which stains F-actin) to identify spines along the neurites (Fig. 1). Images of at least 2 bits of tertiary neurites from each neuron were captured prior to automated analysis using Neurolucida. The total number of spines represent both mature spines, such as mushroom and stubby spines as also the immature thin spines. Phalloidin staining showed decreased spine density in primary neurons from APP/PS1 mice at DIV-15 (Fig. 1 (Bottom)) and the loss was sustained till the last observed time-point of DIV-21. Lower spine density in the primary neurons from APP/PS1 mice indicates a possible reduction in the number of potential synapses or connections that a neuron can form.

### Loss of connectivity in neuronal networks due to synapse loss

The experimental evidence indicates that local changes occur in the brain, at the level of spines. These changes can lead to impaired memory and other behavioral changes when they tend to cause variations in the overall behavior of the collection of interacting neurons. Hence in our model, we use the framework of complex networks to translate the changes at the neuronal level to the level of cognitive functions and memory.

**Figure 2:**
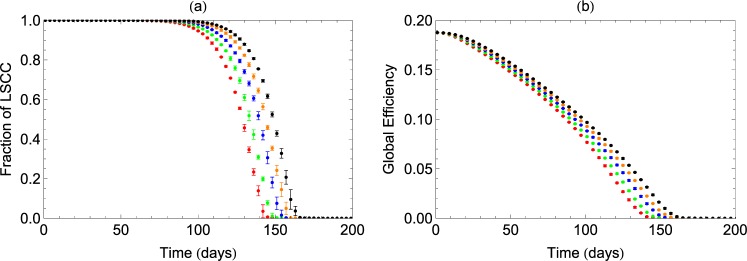
Decrease in the fractional size of LSCC (**a**) and Global Efficiency (**b**) with the loss of synapses or strength of connections in the network. Decay of links is studied for values of *τ* = 20, 25, 30, 35, 40 (left to right). We observe that changes in *τ*, that can take care of variations in individual patient responses or various animal models, give qualitative similar behaviour of LSCC. However, higher values of *τ* indicating slower decay, result in higher value of critical time *Tc*.

To analyze how such losses affect the coordinated activity and efficiency of signal transmission in an assembly of neurons, we study variations in network measures like connectedness and efficiency of transmission that can then characterize the progress of the disease in time. We consider a weighted and directed network at the neuronal level with neurons and synapses constituting the nodes and directed links respectively. Our model network is thus in concurrence with the results of high-clustering (≈0.4) and small-world nature of brain networks reported earlier^27,33,34^. The effects of degradation of synapses, including the possibility of repair and growth, are modelled as an average decrease in healthy synapses leading to loss of connectivity between neurons. The probability *p* with which links are affected by synapse loss is initially taken to be *p*_0_ (taken as 0.01 in our calculations) with a small increase *p*_1_ (taken as 0.0001) at every time step, that takes care of the net increase due to loss and repair, with loss dominating repair or regeneration. The set of links thus chosen randomly with *p*, are affected by synapse loss and their coupling strengths therefore begin to decrease. Assuming an exponential decrease in coupling strengths characterized by a rate *τ*, we designate the links as non-functional when their strength falls to 1/*e* of its initial value. Using inputs from experimental study on synapse loss in APP/PS1 mice presented above, we take *τ* in the range of 20–40 days. The consequent changes in the connectivity of the network, as reflected in the size of the Largest Strongly Connected Component (LSCC), are analyzed progressively in time, to understand the progress of AD. The results of our study are shown in Fig. 2a for different values of the rate of loss.

We note that the fraction of LSCC decreases considerably in 140–160 days for values of *τ* in the range 20–40 days. We repeat the study by varying the value of the initial probability *p*_0_. As *p*_0_ is increased to 0.1 corresponding to much wider spread of synapse loss initially, the behaviour is qualitatively similar but the progress of connectivity loss is faster and LSCC decreases to zero in 50 days for *τ* = 30.

Even while the network remains connected in the initial phase, the loss of spines can affect the strength of coupling and hence the efficiency to transmit signals. To study such changes in the transmission efficiency of the network, we define a measure of global efficiency (GE), as an average measure over the paths having minimum weights between every pair of neurons in the network. The decrease in its value due to loss of spines is shown in Fig. 2b.

**Figure 3:**
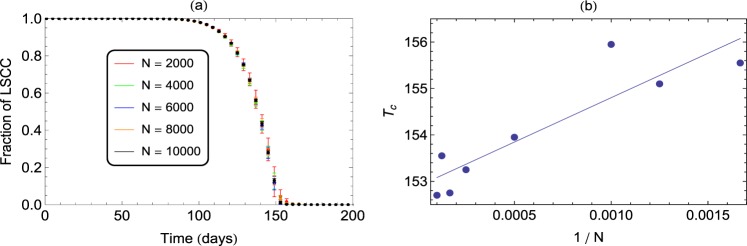
(**a**) Decrease in fractional size of LSCC as a function of loss of spines is studied for different values of network size *N*. We observe that the critical time at which the networks breakdown appears to converge to a critical time *T*_*c*_ as *N* increases. (**a**) Scaling in the behaviour of LSCC with *N* near the transition obtained by plotting *T*_*c*_ values against (1/*N*). The intercept on the y-axis gives the value of *T*_*c*_ in the large size limit as ≈150 days and the slope gives the value of scaling index *β* as 1.0. The results shown for networks of size *N* = 10^4^ and link density of 10 are averaged over 100 realizations.

From Fig. 2a, it is clear that LSCC shows an initial slow decay, corresponding to the knee region of the curve. This is followed by a rapid disintegration that leads to a stage, where the LSCC does not span the whole network, resulting in disintegration of the network. This suggests that the progression of AD can be modelled as a critical phenomenon, where the structural connectivity in the brain undergoes a phase-transition from initial robustness to irreparable breakdown. Thus, we define a critical time *T*_*c*_, as the time beyond which the LSCC ceases to exist, and is identified as the percolation threshold considered in phase transitions. The nature of the transition depends on the size N of the network, as shown in Fig. 3a for identical weights and *τ* = 30. For any N, the fraction of LSCC near the transition, varies with time T as a power-law given by *f* = (*T* − *T*_*c*_)^*β*^. From the log-log plot of *T*_*c*_ versus 1/*N* (Fig. 3b), the value of *T*_*c*_ in the large *N* limit is obtained as the intercept at 1/*N* → 0. Thus, we can estimate the value of *T*_*c*_, applicable to large networks of neurons. For the parameters chosen in our model calculation, we get this critical time as ≈150 days. Also the value of the exponent *β* from the same plot comes out to be 1.0, which agrees with the mean-field value obtained from percolation theory for high-dimensional systems^35^.

**Figure 4:**
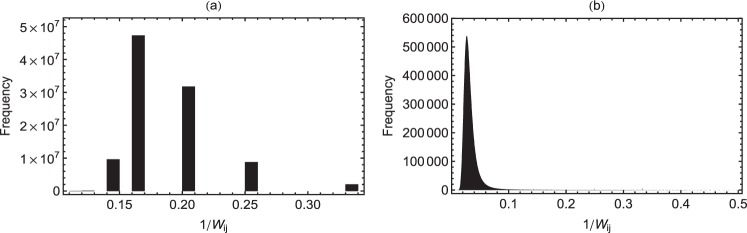
(**a**) Number of paths of maximum-efficiency (or minimum-weight) at the initial stages of connections in the network. In the initial stage, the network has ≈10^7^ directed paths with efficiency in the range 0.2–0.3. (**b**) Final stages of decay after 120 days. With the progressive loss of links, the total number of paths as well as the average efficiency of individual paths decreases.

We compute the counts of the paths of maximum efficiency estimated from inverse of the minimum weights, i.e. the initial value of 1/*W*_*ij*_. (The details of the calculations are given in Methods section). For a typical case with identical weights the counts are shown in Fig. 4a. It shows that before onset of the disease, the number of paths of minimum weights or maximum efficiency is very large (≈10^7^ with efficiency in the range 0.2–0.3). This means multiple paths of highest efficiency are available and hence alternate paths or desired paths for any pattern of activity are available. This structural plasticity corresponds to normal functioning of the brain. However, as shown in Fig. 4b, this count decreases rapidly after ≈120 days, leaving fewer possible paths (≈10^5^) with much lower efficiency (≈0.05). This means that, as the disease progresses, the loss of spines and therefore the functional links, leads to rapid reduction in the total count of highly efficient paths in the network. Thus loss of structural plasticity and disintegration of neuronal networks are both evident during the progress of AD. This will have direct adverse implications on the efficiency of signal transmission in the network of neurons.

**Figure 5:**
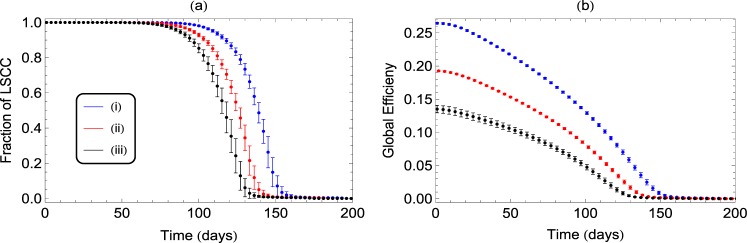
Decrease in fractional size of LSCC (**a**) and Global Efficiency (**b**) for various types of initial distributions of synaptic coupling strengths. (i) identical coupling strengths (ii) normal distribution and (iii) log-normal distribution.

In order to model a more realistic assembly of neurons, where the initial weights of connections need not be identical for all neurons, we consider two different distributions for initial weights. Considering studies reported earlier^36,37^, we take a normal distribution of weights with mean 0.7 and standard deviation 0.2 and a log-normal distribution with mean 4 and standard deviation 0.5 and rescaled to the interval (0,1). The results averaged over 100 realisations, on networks with 1000 nodes and average degree 10, are compared with that from identical weights in Fig. 5 for *τ* = 30.

### Loss of connectivity due to synapse loss in networks from human brain network data

**Figure 6:**
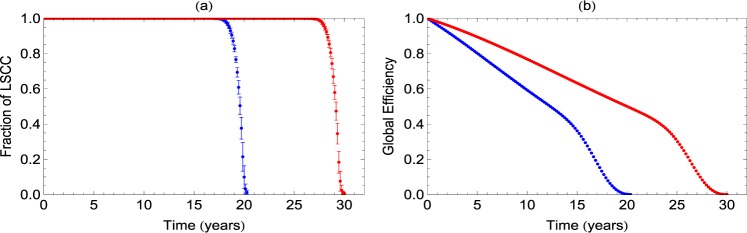
Decrease in the fractional size of the LSCC (**a**) and Global Efficiency (**b**) with synapse losses in the constructed human brain network. Decay of links is studied for values of *τ* = 40 (blue) and *τ* = 80 (Red). The values of GE are normalized by the initial value to emphasize the relative decrease due to synaptic decay.

The results reported so far are based on studies on a network model derived from observations on cultured neurons from APP/PS1 mice. We now apply the same analysis to human brain network data derived from the Van Essen parcellation. We use the mesoscopic network of the human brain constructed by putting together data of 2 subjects drawn from the HCP database^38,39^. With the value of parameter *p*_0_ as 0.004 and *p*_1_ as 10^−6^, we repeat the study for *τ* = 40 and *τ* = 80. The results are shown in Fig. 6. We observe that the LSCC shows very strong and extensive robustness to decay initially but beyond a critical time, it undergoes rapid deterioration. This is due to the vast number of redundant connections present in the human brain that prevent immediate structural breakdown. For *τ* = 40, breakdown happens at ≈20 years and for *τ* = 80, it is around 30 years. While the behaviour of LSCC does not reveal the immediate effects of synaptic decay and loss, the changes are evident earlier in behaviour of GE. The decrease of GE shows two regions, with two different slopes. The initial smaller slope is indicative of the increasing path-lengths (or lowered efficiency) of existing paths. During this time, the LSCC is still very robust and spans the entire network. During the later stages of decay, the slope abruptly increases. This is the point where the network begins to fall apart and the disconnected components begin to appear and loss of connecting paths dominates over loss of efficiency of existing paths.

## Discussion

In this study we show that spine loss/disassembly occurs in primary neurons cultured from AD transgenic mice (APP/PS1) at DIV 15 and continues till the culmination of the experiment at DIV 21. These changes that happen at the individual neuronal level start affecting the brain functions when they translate into consequent changes in the collective response of a collection of interacting neurons. We present a mathematical model that incorporates the above experimental observation into the framework of complex networks. We show how the disassembly of spines leads to degeneration of the network, that can be understood as a critical phase transition and gives an estimate of the time for complete degeneration or impairment. Further, our model also shows that there is substantial decrease in the number of efficient alternate paths which could potentially result in loss of efficient signal transmission. The study thus clearly shows how a reduction in synaptic density can lead to loss of connectivity and decrease in efficiency and number of multiple paths and eventually over time, to a total disintegration of the neuronal network.

In this context, we mention that graph theory based studies have been reported earlier, using functional connectivity derived from EEG in 15 Alzheimer patients and 13 control subjects^27^. While the above study invokes networks derived from external observational data, in our study, we present measures on networks directly at the level of neurons derived from *in vitro* experiments on cultured neurons from APP/PS1 mice.

We primarily focus on the extent of connectivity in the network and its efficiency for transmission of signals as important measures that can keep track of the progress of degeneration occurring in time. In this case, we find that the progress of the disease can be traced from the fraction *f* of nodes belonging to LSCC and the efficiency and count of links with high efficiency. We find that *f* decreases by 10% in 4 months and completely vanishes over 5 months. These results, on an average, agree with cognitive test impairments reported on transgenic mouse models of AD^40,41^.

We also monitor the changes in the path-length and clustering coefficient (CC) and find reduction in CC by 25% and an increase in path length to 7.35 from 5.5 at the onset of AD. This is in agreement with the study based on EEG where the path length is found significantly larger for the Alzheimer group compared with the control group^27^. It is interesting that similar results are obtained from our studies at the neuronal networks level also.

We also illustrate the application of our analysis to human brain network data derived from the Van Essen parcellation. In this case, we find that in the initial stages, the LSCC shows robustness to decay but GE shows slow decay, indicating the existence multiple paths of increasing path-lengths and hence lowered efficiency. Beyond a critical time of 20–30 years, there is rapid deterioration.

The network model considered here is fairly simple and further details and specific variations can be built into it to make more accurate predictions. We illustrate this by repeating the study for normal and log normal distributions of initial synaptic weights for neurons. Moreover, the model is very general with three tunable parameters, the timescale parameter *τ*, initial probability of synapse loss *p*_0_ and rate of increase in probability *p*. The calculations in the present study are done with these parameters derived from the observed data from experiments. Our results already include a range of values of the time scale parameter *τ*, 20–40 days that gives the time of disintegration of networks as 140–150 days for the mice and *τ* = (40, 80) gives the critical time as 20–30 years from human brain network data. We repeated the analysis with varying initial probability *p*_0_, which would imply varying spread of spine losses, and find that larger *p*_0_ leads to faster decrease in connectivity. Thus, the heterogeneity of individual patient responses or various biological parameters from animal models can be incorporated easily with appropriate choice of these parameters.

The present study could also be used to predict the progression of dementia using FDG utilization seen by PET imaging carried out longitudinally in human subjects. This may be particularly useful in patients harbouring familial mutations for AD. Development of such models using longitudinal data provides mechanistic insights and will be predictive of the future course of the disease.

## Methods

### Primary cortical cultures

Transgenic mice B6C3-Tg (APPSwe/PS1ΔE9)85Dbo/J (https://www.jax.org/strain/005864)^42^ were obtained from Jackson Laboratory, USA and bred at the institutional Central Animal Facility. All animal experiments were carried out in accordance with institutional guidelines for the care and use of laboratory animals under approval of the Institutional Animal Ethics Committee, Indian Institute of Science, Bangalore. Efforts were made to reduce the suffering of animals and the numbers used. Cortical tissue was dissected out from P1 APP/PS1 and wild-type littermate mice. All pups were genotyped to confirm the presence or absence of the transgene. Primary neuronal cultures were established and maintained according to previously mentioned protocol^43^. Briefly, cortical tissue was dissected out and trypsin was used to enzymatically dissociate the tissue. The tissue was then triturated to obtain a homogeneous cell suspension which was centrifuged at 800 rpm. The pellet was resuspended and plated in Neurobasal-A Medium (GIBCO, Life Technologies, CA, USA) containing 1% 100X Glutamax (GIBCO, Life Technologies, CA, USA), 1% 100X penicillin-streptomycin (GIBCO, Life Technologies, CA, USA) and 2% 50X B27 (GIBCO, Life Technologies, CA, USA). Cells were plated on cover slips precoated with poly-D-lysine (0.1 mg/ml) and maintained at 37 °C in 5% CO_2_ in serum-free condition, as described above. Primary cortical neurons at four different time points (DIV 5, DIV 10, DIV 15 and DIV 21) were fixed with 2% paraformaldehyde (w/v) and labeled with Acti-Stain 488 phalloidin (catalog #PHDG1, Cytoskeleton, USA). The coverslips were mounted with an antifade mounting medium and used for confocal imaging.

### Imaging and Image analysis

Carl Zeiss LSM780 laser scanning microscope was used to acquire the confocal images. Argon laser 488 was used to capture Actin stained with phalloidin 488. The images were acquired with an oil immersion objective 63X/1.4 NA. Equal bits of tertiary neurites (minimum of 2 neurites per neuron) were captured with 512 × 512 frame size, pixel size of 90 nm, a zoom factor of 3,12 bit depth, pinhole 1 (A.U) Airy Unit and an optimal z-Interval of 0.4 nm. The z-stack images were loaded onto Neurolucida 360 (MBF sciences). Maximum intensity projections (MIPs) were generated for spine detection and quantification. The dendrites were traced along the backbone of the dendrites in the 3D mode. After the tracing, spines were automatically detected using the, *Spinedetectionmode*. Average spine density was generated by the Neurolucida explorer software for every dendrite. The confocal images were acquired and analyzed under the above mentioned conditions after blinding.

### Statistical analysis

Graphs for all image analysis data were prepared in GraphPad Prism. Two-way RM ANOVA was used for statistical analysis. Results are represented as mean ± standard error of the mean (SEM) and after Bonferroni post-hoc test, *p* values < 0.0001 were regarded as significant.

### Network construction

A directed network of small world nature is generated with 10^4^ nodes and 10^5^ links, average clustering coefficient of ≈0.4 and path length of ≈5.5, in concurrence with the recently reported results on brain networks^27,33,34^. We are interested in the transition from a completely healthy set of synapses to a configuration where the synapses have decayed to such an extreme extent (on an average) that no communication is possible between any 2 neurons. So we initialize all the edge-weights to 1 (assuming 1 represents a healthy state and 0 a decayed state). This allows us to study the performance of synapses w.r.t their own early healthy states. However, once the process of decay sets in, different fractions of synapses decay at different times and relative distribution of edge-weights sets in.

### Human brain network dataset

The network of the human brain was constructed using data derived from the Van Essen parcellation. The connectivity matrix was put together using data from 2 subjects drawn from the HCP database^38,39^. This network is organized at the mesoscopic level where each node represents a region in the human brain and edges are the interaction between these regions with 360 nodes with 43516 directed and weighted edges where the weights represent the strength of interaction. Although the originally constructed network contains self-loops, we remove them for the purpose of this study. The resulting network is extremely dense, has very high clustering and very small path-length.

### Loss of connectivity

The loss of connections in the networks occurs due to spine-decay or loss of healthy spines caused by A*β* and this is modelled as an exponential reduction in the coupling strengths of the links between any two neurons. The probability *p* with which links are affected by synapse loss is initially taken to be *p*_0_ (taken as 0.01 in our calculations) with a small increase *p*_1_ (taken as 0.0001) at every time step. This increase will be the net increase due to loss and repair with the loss dominating the repair or regeneration. This results in a certain number of randomly chosen links getting affected and their coupling strengths therefore begin to decrease. It is clear that a higher value of *p*_0_ indicates that the rate of spread of the disease is higher, resulting in larger number of healthy synapses getting affected as time progresses. The gradual increase of *p*_0_ that is built into the model through *p* is designed to capture the progressive worsening of the disease.

We take the decrease in coupling strength *ε*_*ij*_, between neurons *i* and *j*, to be exponential in a time-scale set by parameter *τ* as *ε*_*ij*_ = *ε*_0_.*exp*(−*t*/*τ*). This change in coupling strength continues in time as the disease advances. When it falls to 1/*e* of its initial value, the link is assumed to be totally non-functional and hence removed from the network. The total strength of coupling of all links in the network is the quantity that can be directly correlated with the average spine density of the whole network. It is clear from the experimental observations presented, that the spine density decreases by 50% in 3 weeks. In accordance with this, we use the time scale of decay *τ* within the range of 20–40 days, which would result in approximately the same amount of decay in 21 days.

### Largest strongly connected component

The Largest Strongly Connected Component (LSCC) is the set of all nodes in the network for which there exists a directed path from every node to every other node. The normalized size *f* of the LSCC is defined as the fraction of nodes in the full network that belong to LSCC. It gives the largest fraction of nodes in the network that can be reached through any transmission process and is therefore a measure of the extent of relevant connectivity in the network. When all nodes in the network belong to LSCC, the normalized size *f* of the LSCC is 1. Then, every neuron in the network can be reached from every other neuron, though the path may not be direct via a single link but instead a concatenation of multiple links involving many intermediate neurons. This implies that a signal reaching any given neuron can be transmitted (if required) to all other neurons and in particular, to the desired target neuron. Hence the target neuron will receive all inputs required to cross its activation threshold and therefore can generate any required activity pattern. On the other hand, *f* < 1 corresponds to a situation where a fraction of neurons, even though not totally isolated, cannot transmit signals to a target neuron potentially leading to a situation where the integrated input to that neuron is less than the activation threshold. Consequently, distortions could arise in the expected activity pattern of the target neuron.

### Estimation of critical time *T*_*c*_

To characterize further the loss of connectivity due to synapse loss, we treat the progression of AD as a critical phenomenon with structural connectivity undergoing a phase-transition at this point. This transition point is identified using percolation theory^35,44–47^, which is well-studied in the context of phase transitions. This provides methods to study the behaviour of the order parameter, as a function of a tuning parameter, particularly in the vicinity of the critical value of the tuning parameter. This gives insight into the scale-free behaviour of the order parameter near the critical point and is characterized by the scaling exponents. In our problem, the fractional size of the LSCC is the order parameter and time is the tuning parameter. In the region close to the critical point, we rescale the axis so that the critical point becomes the new origin and fit the behaviour of LSCC with a power law function of |*T* − *T*_*c*_|, where *T*_*c*_ is the critical time and *β* is the scaling exponent. Though we do not know the exact value of *T*_*c*_, we consider the approximate region where *T*_*c*_ may lie and then plot LSCC vs (*T* − *T*_*c*_)^*β*^, for various values of *T*_*c*_. In this case, as we go further away from *T*_*c*_, the value of LSCC increases and therefore the value of the exponent is expected to be positive *β* = 1. For each value of *T*_*c*_, we find the best fit (in terms of the *R*^2^ value) is seen for *β* = 1^35^. For that value of N, with *β* = 1, the value of *T*_*c*_ for which the *R*^2^ is maximum is selected. This process is repeated for different values of N. Then, we plot *T*_*c*_ vs 1/*N*, to estimate the value of *T*_*c*_ asymptotically for 1/*N* going to 0 (or equivalently *N* → ∞). This is then presented as the final value of the critical time in the large N limit, which in our case was found to be ≈150 days for the network model with corresponding value of *β* as 1.0.(Fig. 3b).

### Global efficiency

Associated with every link between neurons *i* and *j* in the network, is a probability of connection (as described earlier) that can be taken as its coupling strength or conductance. The inverse of this coupling strength is defined as the weight of the link which measures the resistance to transmission. The weight of a path from a neuron *i* to another neuron *j* is the cumulative weight of all links constituting the path from *i* to *j*,

1 $${w}_{ij}={w}_{i{k}_{1}}+{w}_{{k}_{1}{k}_{2}}+\mathrm{...}+{w}_{{k}_{n-1}{k}_{n}}+{w}_{{k}_{n}j}$$

where *k*_1_ to *k*_*n*_ correspond to neurons on the path connecting *i* to *j*. For each pair (*i*, *j*) in the network, among all the paths connecting them, the path of minimum weight is found and its weight is taken as *w*_*ij*_. This path will then correspond to the one with maximum conductance or efficiency for transmission and 1/*w*_*ij*_ will be a measure of this efficiency. The average of 1/*w*_*ij*_ over all such possible paths of least weight in the network is defined as the global efficiency (GE)^26^.

2 $$GE=\frac{1}{N}\sum _{i=1}^{N}\frac{1}{N-1}\sum _{j=\mathrm{1,}j\ne i}^{N}\frac{1}{{w}_{ij}}$$

At every time-step, corresponding to 1 day, the two measures mentioned above, *f* and GE are calculated and their average values over 100 realizations of the networks are presented in the results. The counts of paths having different efficiency are also calculated using above approach. Clearly large counts (≈5*x*10^7^ here) with very large efficiency are indicative of a high level of plasticity as in the healthy brain. However due to loss of spines these counts come down drastically as AD progresses, so that the possibility of choosing alternate paths or desired paths for any pattern of activity becomes very low.

### Non-Uniform distribution of initial synaptic coupling strengths

Throughout the study, we have worked with the case where the initial network is constructed with identical synaptic weights (=1). This is because we are interested in studying the change in coupling strength of a synapse w.r.t its own healthy state. We obtain qualitatively similar results for two cases of non-uniform distributions of initial weights. From reported results^36,37^, we choose these distributions as normal distribution with (*μ*, *σ*) = (0.7, 0.2) and a log-normal distribution with (*μ*, *σ*) = (4, 0.5) rescaled to the interval (0, 1), where *μ* and *σ* denote the mean and standard deviation of the distributions.
